# Personal and Ambient Air Pollution Exposures and Lung Function Decrements in Children with Asthma

**DOI:** 10.1289/ehp.10911

**Published:** 2007-11-22

**Authors:** Ralph J. Delfino, Norbert Staimer, Thomas Tjoa, Dan Gillen, Michael T. Kleinman, Constantinos Sioutas, Dan Cooper

**Affiliations:** 1 Department of Epidemiology, School of Medicine, University of California, Irvine, Irvine, California, USA; 2 Department of Statistics, School of Information and Computer Sciences, University of California, Irvine, Irvine, California, USA; 3 Department of Community and Environmental Medicine, School of Medicine, University of California, Irvine, Irvine, California, USA; 4 Department of Civil and Environmental Engineering, School of Engineering, University of Southern California, Los Angeles, California, USA; 5 Department of Pediatrics, School of Medicine, University of California, Irvine, Irvine, California, USA

**Keywords:** asthma, epidemiology, forced expiratory flow rates, longitudinal data analysis, nitrogen dioxide, panel study, particulate air pollution

## Abstract

**Background:**

Epidemiologic studies have shown associations between asthma outcomes and outdoor air pollutants such as nitrogen dioxide and particulate matter mass < 2.5 μm in diameter (PM_2.5_). Independent effects of specific pollutants have been difficult to detect because most studies have relied on highly correlated central-site measurements.

**Objectives:**

This study was designed to evaluate the relationship of daily changes in percent-predicted forced expiratory volume in 1 sec (FEV_1_) with personal and ambient air pollutant exposures.

**Methods:**

For 10 days each, we followed 53 subjects with asthma who were 9–18 years of age and living in the Los Angeles, California, air basin. Subjects self-administered home spirometry in themorning, afternoon, and evening. We measured personal hourly PM_2.5_ mass, 24-hr PM_2.5_ elemental and organic carbon (EC–OC), and 24-hr NO_2_, and the same 24-hr average outdoor central-site(ambient) exposures. We analyzed data with transitional mixed models controlling for personal temperature and humidity, and as-needed β_2_-agonist inhaler use.

**Results:**

FEV_1_ decrements were significantly associated with increasing hourly peak and daily average personal PM_2.5_, but not ambient PM_2.5_. Personal NO_2_ was also inversely associated with FEV_1_. Ambient NO_2_ was more weakly associated. We found stronger associations among 37 subjects not taking controller bronchodilators as follows: Personal EC–OC was inversely associated with morning FEV_1_; for an interquartile increase of 71 μg/m^3^ 1-hr maximum personal PM_2.5_, overall percent-predicted FEV_1_ decreased by 1.32% [95% confidence interval (CI), −2.00 to −0.65%]; and for an interquartile increase of 16.8 ppb 2-day average personal NO_2_, overall percent-predicted FEV_1_ decreased by 2.45% (95% CI, −3.57 to −1.33%). Associations of both personal PM_2.5_ and NO_2_ with FEV_1_ remained when co-regressed, and both confounded ambient NO_2_.

**Conclusions:**

Independent pollutant associations with lung function might be missed using ambient data alone. Different sets of causal components are suggested by independence of FEV_1_ associations with personal PM_2.5_ mass from associations with personal NO_2_.

Acute adverse effects of air pollution on asthma outcomes in small cohorts of children have been reported in longitudinal studies using repeated daily measurements (panel studies). More recently, this includes positive associations between a biomarker of airway inflammation, exhaled nitric oxide, and both personal and outdoor ambient air pollutant exposures in children with asthma (Delfino et al. 2006; Koenig et al. 2005). Most panel studies of daily air pollution and acute changes in expiratory lung function reported before 2004 used measurements of peak expiratory flow (PEF). They generally showed consistent, albeit heterogeneous, inverse associations of PEF with ambient particulate matter (PM) < 2.5 μm in diameter (PM_2.5_), with somewhat weaker associations for PM < 10 μm in diameter (PM_10_) [reviewed by Ward and Ayers (2004)]. However, PEF is more effort dependent than another measure of lung function, forced expiratory volume in 1 sec (FEV_1_). PEF is also a poor surrogate of the more clinically relevant FEV_1_ (Giannini et al. 1997; Thiadens et al. 1999) because PEF measures only the first portion of expiration from larger proximal airways, whereas FEV_1_ reflects resistance in both proximal and distal airways. Lower FEV_1_ occurs when flow rate decreases because of airway obstruction, which is a key phenotype of asthma.

Most previous studies of the relationship between acute asthma in children and air pollution have relied on ambient central-site data (Sarnat and Holguin 2007; Trasande and Thurston 2005). Exposure error from using this data will likely diminish the accuracy of exposure–response estimates. High interpollutant correlations at ambient monitoring sites also make it difficult to identify independent associations from different regulated criteria air pollutants such as PM_2.5_ and nitrogen dioxide. Furthermore, criteria pollutants may be serving as markers for components not routinely monitored, such as combustion-related organic compounds. These component mixtures may lead to airway inflammation and bronchoconstriction. However, a range of individual responses for a given type of component exposure is likely for children with asthma. Children at greatest risk likely include those with persistent asthma, particularly if they are not taking controller medications. Personal exposure assessments (Jerrett et al. 2005) and assessments of clinical and biological differences in an individual’s asthma (Sarnat and Holguin 2007) have been proposed to clarify these issues regarding exposure and response.

We previously found that associations of asthma symptoms with ambient PM mass concentrations were completely explained by ambient elemental and organic carbon fractions of PM (EC and OC, respectively) (Delfino et al. 2003). Studies have shown much stronger correlations between traffic emission sources and EC (or a similar measure of black carbon reflectance) compared with PM mass (Cyrys et al. 2003), but OC is more difficult to apportion to emission sources (Fujita et al. 2007). Our earlier finding thus suggested that products of fossil fuel combustion were important in asthma outcomes that might otherwise be ascribed to uncharacterized PM mass. Many studies have also shown strong correlations between traffic emission sources and NO_2_ (Jerrett et al. 2005). In another panel study, we reported positive associations between repeated measures of exhaled NO and personal exposures to NO_2_ and EC that were largely independent of associations with personal PM_2.5_ mass (Delfino et al. 2006). These findings suggested that in addition to products of fossil fuel combustion, other particle components in personal air samples were proinflammatory. Here we aim to expand on these previous findings in the same cohort of children by evaluating the relationship of FEV_1_ to both personal and central-site NO_2_, PM_2.5_ mass, and EC–OC fractions of PM_2.5_.

## Materials and Methods

### Design and population

We followed a panel of 63 schoolchildren with asthma for daily repeated measures of personal exposure to air pollution in two regions of the Los Angeles air basin in Southern California: Riverside and Whittier. These regions are characterized by high levels of air pollution predominantly from mobile sources of fossil fuel combustion. Geographic areas of recruitment were delimited to a 10-mile radius around a central air monitoring site in Riverside (population density, 3,538/mi^2^), and a 5-mile radius around a central air monitoring site in Whittier (5,947/mi^2^) (RAND California 2007). The institutional review board of the University of California, Irvine, approved the study protocol. We obtained informed written consent from all subjects and one of their legal guardians.

We recruited subjects by referral to the study office by local school district nurses. Eligibility criteria included ages 9–18 years and parent-reported physician-diagnosed asthma, with a history of episodic symptoms including wheezing, cough, or dyspnea. For the cohort, we targeted children with evidence of mild to moderate persistent asthma, including *a*) a history in the previous 12 months of asthma exacerbations requiring the use of prescribed bronchodilator(s) on ≥ 2 days per week, regardless of anti-inflammatory medication use; *b*) current use of oral or inhaled anti-inflammatory medications, regardless of symptom frequency; or *c*) < 80% predicted normal FEV_1_ from office spirometry at the subject’s baseline visit to the General Clinical Research Center, University of California, Irvine. Subjects were ineligible if they smoked or if someone smoked in the subject’s home.

We followed subjects daily over a continuous 10-day period that involved wearing air samplers to measure personal exposure to air pollutants. There were sixteen 10-day periods of follow-up (a run) from July to December 2003 (Riverside) and 2004 (Whittier). Four subjects were followed daily at their home in each 10-day run (except one run with three subjects).

### Lung function and diary data

We have presented spirometry methods and validation results for the present panel subjects in detail in our previous report (Thompson et al. 2006). Subjects self-administered spirometry at home using the hand-held ndd EasyOne Frontline Spirometer (ndd Technologies, Chelmsford, MA). Subjects were given detailed instructions and trained on its use in the home during a 5-day run-in period. Subjects were instructed to perform spirometry in the morning (up to 1100 hours), afternoon (1500–1800 hours), and evening (2000–2400 hours), referred to here as “session period.” Subjects were also instructed to complete a personal digital assistant (PDA) diary every 2 waking hours reporting asthma medication use. We mitigated missed PDA diary prompts with paper diaries and daily technician-administered questionnaires. Medications reported included daily preventive (controller) medications and as-needed (rescue) medications (inhaled β_2_-agonist bronchodilators). Near bedtime, the PDA diary prompted recall of rescue and controller medication use throughout the day. In the morning, it prompted recall of rescue inhaler use during the night. We also measured rescue inhaler use with a pressure-actuated recording device (Doser; Meditrack Products, Hudson, MA) that logged puffs in 24-hr intervals from midnight to midnight.

During each session, the spirometer stopped after three good spirometry maneuvers were obtained, and it gave each subject up to six chances to meet acceptability and repeatability criteria. Intermittent instructions to subjects were displayed on the spirometer’s display based on the success or type of error of each attempt. Subjects were instructed to perform sessions before the use of inhaled β_2_-agonist bronchodilator medications unless necessary, and to wait at least 4 hr after the use of them before performing a session. At the end of spirometry maneuvers, subjects answered a yes/no question on the spirometer screen: “Did you need to use your rescue medication in the last hour?”

Research technicians downloaded the spirometry data into laptops during daily home visits, and checked compliance and acceptability of maneuvers as generated by the ndd software (version 2.6). We retrained subjects as needed. Compliance was enhanced by monetary incentives, an on-screen point system, and audio alarms. We later evaluated each curve for acceptability and repeatability by selected criteria as previously described (Thompson et al. 2006). We then further evaluated these curves for visual acceptability. We found compliance was high (94%) and the number of sessions with acceptable and reproducible maneuvers by objective criteria as well as visually acceptable was moderately good (69%) (Thompson et al. 2006). To ensure a suitably complete time series of repeated measures, subjects included in the present analysis had to have at least a third of their 29 expected FEV_1_ maneuvers over the 10 days that were valid as such. We excluded 10 subjects who did not meet this compliance threshold, leaving 53 subjects who had 1,249 observed of 1,537 expected spirometry sessions (81%) with acceptable and reproducible maneuvers (individual subject range, 41–100%, median 86%).

The highest FEV_1_ (best effort) from the two acceptable and reproducible maneuvers was selected for analysis. We analyzed percent-predicted normal FEV_1_ based on a subject’s height, age, sex, and race/ethnicity (Hankinson et al. 1999). This standardizes measurements between subjects, provides overall estimates of association for the study population, and is clinically meaningful.

### Exposures

The personal air monitors were active air samplers worn in a backpack daily over the 10 consecutive days. Personal measurements included continuous nephelometer mass measurements of PM_2.5_ (personal DataRAM model 1200; MIE Inc., Bedford, MA) and 24-hr EC and OC fractions of PM_2.5_, collected on quartz filters (Whatman Inc., Florham Park, NJ) using an attached filter cassette. A 2.5-μm sharp-cut cyclone was attached upstream of the nephelometer, and PM_2.5_ for EC and OC was collected down-stream at a flow rate of 4 L/min. We measured NO_2_ over 24-hr periods using a miniaturized diaphragm pump (VMP1625; Virtual Industry, Colorado Springs, CO) run at 0.1 L/min to sample air through tri-ethanolamine-treated molecular sieve sorbent tubes (SKC West Inc., Fullerton, CA). We measured NO_2_ based on National Institute for Occupational Safety and Health (1994) Method 6014. We collected personal temperature and relative humidity with attached loggers (Onset Computer Corp., Pocasset, MA). Elsewhere we provide data on the validation of both the personal PM_2.5_ sampler (Chakrabarti et al. 2004) and our personal NO_2_ active sampler (Staimer et al. 2005).

We measured a parallel set of exposures at our own outdoor central sites, one in Riverside and one in Whittier. PM_2.5_ and PM_10_ mass (Teflon filters), and PM_2.5_ EC and OC (quartz filters) were collected there using standard procedures with Harvard Impactors (Air Diagnostics and Engineering, Inc., Naples, ME). Sampling start and stop times occurred during the early evening of each day near the same time as personal samplers. For both personal and central-site sample collection on quartz filters, particulate carbon was speciated into OC and EC using the thermal manganese dioxide oxidation technique (Fung et al. 2002). Central-site gases included hourly ozone and NO_2_ measured by the South Coast Air Quality Management District. In Riverside, the district site was centrally located, and we sited Harvard Impactors there. In Whittier, we constructed a central site at a subject home elevated on a hill. However, data for O_3_ and NO_2_ came from two district sites at opposite ends of the Whittier study region. We averaged hourly concentrations of these gases for the two stations.

### Analysis

We tested the relationship between percent-predicted FEV_1_ and each air pollutant using linear mixed-effects models, with each subject serving as his or her own control (Verbeke and Molenberghs 2001). Because correlation among outcomes was present for the within-individual repeated measures, and possibly for the exposure run, we assumed a two-stage hierarchical model with random effects at the subject level, nested within a run. We fit an autoregressive-1 correlation structure given the observed variability from empirical variograms. Air pollutant exposures were mean-centered by subject to yield comparability between subjects and across runs (Sheppard et al. 2005).

We investigated impacts of personal hourly PM_2.5_ mass exposures preceding the FEV_1_ measurement including the average of the preceding 24 hr (lag 0), the average of the 25th through 48th hr (lag 1), and a cumulative 2-day moving average. We retained PM_2.5_ data if at least 75% of the hours were nonmissing. The same approach was used for central-site hourly NO_2_. Given our previous findings (Delfino et al. 1998, 2002), we also examined 1-hr and 8-hr maximum moving average in personal PM_2.5_ during the 24 hr preceding the FEV_1_ measurement. We examined 8-hr peak central-site O_3_ given its well-known diurnal trend. For the filter-based measurements (personal and central-site EC and OC, and central-site PM_2.5_ mass) and for personal NO_2_, we defined lag 0 to be the same day and lag 1 was the preceding day’s 24-hr measurement. We did not extend the number of lags beyond that last 2 days to maintain a reasonable within-subject sample size, because a subject’s data were limited to a single 10-day consecutive monitoring period. We expressed results as percent change in predicted FEV_1_ per interquartile range (IQR) increase in each pollutant to standardize inter-pollutant comparisons.

We fit transitional models by adjusting for the previous FEV_1_ measurement to control for observed sinusoidal circadian rhythms. Transitional models condition the outcome in the current time on the previous outcome observation (e.g., afternoon FEV_1_ is regressed on the morning FEV_1_) (Diggle et al. 2002). We also tested for effect modification by session period (morning, afternoon, evening), and found several differences that we present below.

We decided *a priori* to adjust for use of rescue inhalers, including use last night, which was associated with a decrease of 3.5 percent-predicted FEV_1_ in the afternoon and evening [95% confidence interval (CI), −6.5 to −0.4]. We also included cumulative daily use of rescue inhalers during the previous day using Doser data [PDA diary data for 119 person-days were used where Doser data were missing (9.5%)]. Cumulative inhaler use was positively associated with an increase of 1.1 percent-predicted FEV_1_ in the morning per two-puff dose (95% CI, −0.2 to 2.5). In addition, we excluded observations where subjects reported use of rescue inhalers in either the ndd spirometer diary or PDA diary report covering the last 2–4 hr (57 FEV_1_ observations, 4.6% of total). Such use was associated with an increase of 2.2 percent-predicted FEV_1_ (95% CI, 0.07 to 4.3). Models also adjusted for personal temperature and relative humidity (both positively associated with FEV_1_).

We tested potential confounding by self-reported respiratory infections (22 person-days, 4.4% of total, *p* = 0.86 in relation to FEV_1_). We also tested confounding by the two regions of study, session period of day (morning, afternoon, or evening), and weekend. None of these variables influenced associations, with one exception discussed below for session period.

We conducted residual diagnostics to assess the presence of influential data points and subject clusters, as well as deviations from assumed functional form. One 10-year-old white female subject influenced personal PM models leading to a decrease in personal PM_2.5_ regression parameter estimates and increase in SE (Cook’s D, 0.38; restricted likelihood distance, 4.41). We present results with this subject and sensitivity analyses removing her data.

Given prior evidence (Becklake and Kauffman 1999; Delfino et al. 1998, 2002, 2006), we further tested models for effect modification by sex and by asthma controller medications using product terms with each air pollutant. We assumed product term interactions with a *p*-value < 0.1 suggested possible effect modification. We tested a binary (yes/no) indicator for use of anti-inflammatory medications, as well as separate indicators for inhaled corticosteroids with versus without leukotriene receptor antagonists. We also tested a binary indicator for prescribed daily use of short- or long-acting bronchodilators as controller medications. We anticipated both controller and rescue bronchodilators to have major impacts on temporal changes in FEV_1_.

We tested two-pollutant regression models to assess between-pollutant confounding after testing interaction between the pollutants in product term models. The aim here was to assess the extent to which associations with one pollutant was independent of another pollutant.

We retested selected regression models using generalized estimating equations with robust standard error estimates (Diggle et al. 2002) as a validity check to likelihood assumptions of the linear mixed-effects model. We found no qualitative differences in our study results.

Finally, we used a fifth-order polynomial distributed-lag mixed-effects model (Schwartz 2000) to investigate the relationship of FEV_1_ to lagged hourly personal PM_2.5_ exposures out to 48 hr. We found negligible difference in the response curves when models that are more flexible were considered. We fit distributed lag models via a linear mixed-effects model assuming an autoregressive-1 correlation structure.

## Results

### Descriptive data

Descriptive statistics for the 53 subjects in the present analysis are presented in Table 1. On average, FEV_1_ was lowest in the morning and gradually increased to its highest in the afternoon, then decreased toward the evening (Table 2). We found percent-predicted FEV_1_ was significantly higher among 28 subjects taking inhaled corticosteroids, and significantly lower among 13 subjects taking antileukotrienes compared with 20 subjects not taking controller medications (Table 2). There was no significant difference in FEV_1_ for 16 subjects taking controller bronchodilators versus those not taking them.

We collected 519 person-days of valid observations for the personal NO_2_ air monitor. It malfunctioned for only 3 person-days. The PM_2.5_ nephelometer malfunctioned for two subjects during most of their 10-day run and periodically for other subjects, leaving 416 person-days of observation. Table 3 presents descriptive statistics for the exposure data. Concentrations of peak hourly personal PM_2.5_ were high, averaging 90 μg/m^3^ with a maximum reaching 603 μg/m^3^. The U.S. Environmental Protection Agency National Ambient Air Quality Standard (NAAQS) for 8-hr ambient O_3_ (80 ppb) was never exceeded (U.S. Environmental Protection Agency 2008). However, the NAAQS for 24-hr average ambient PM_2.5_ (35 μg/m^3^) was exceeded on 28 of 170 days and NAAQS for 24-hr average ambient PM_10_ (150 μg/m^3^) was exceeded on only 1 day at the central sites.

**Figure 1:**
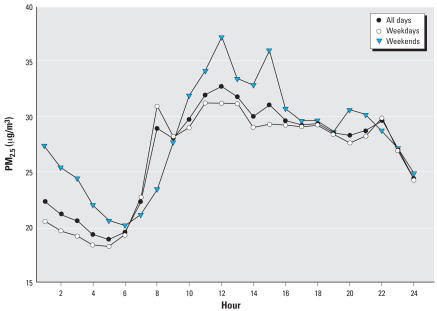
Hourly average concentration of personal PM_2.5_ across 51 subjects for all days, weekdays, and weekends.

Figure 1 shows hourly average concentrations of personal PM_2.5_. Concentrations were lowest in the early morning, abruptly rising mid-morning with maximums around noon and sustained concentrations until late evening. The mid-morning peak occurred around 0800 hr during the school weekday but was delayed by several hours and was higher on weekends.

Table 4 shows the between-pollutant correlations. Small significant correlations of personal PM_2.5_ with personal EC, OC, and NO_2_ were found. Personal PM_2.5_ was moderately correlated with ambient PM_2.5_ (Spearman *r* = 0.60), and had small correlations with personal NO_2_ (*r* = 0.38) and ambient NO_2_ (*r* = 0.32). Personal NO_2_ showed low moderate correlation with ambient NO_2_ (Spearman *r* = 0.43). However, personal EC and OC were not correlated with ambient EC and OC but were weakly correlated with ambient NO_2_. Ambient exposures were moderately correlated with each other.

### Regression analysis

Table 5 shows models for the relationship between percent-predicted FEV_1_ and air pollutants. We found significant inverse associations between FEV_1_ and 1-hr and 8-hr peak personal PM_2.5_ measured over the 24-hr periods preceding the lung function measurements (lag 0). The model for lag 0 24-hr average personal PM_2.5_ showed smaller associations for an interquartile increase in exposure, and was of borderline significance (*p* < 0.08). However, dropping the one influential subject discussed in “Methods” led to a stronger significant association with 24-hr personal PM_2.5_ (−0.69% predicted FEV_1_; 95% CI, −1.34 to −0.04%). Outdoor central-site 24-hr average PM_2.5_ (Table 5) and PM_10_ (not shown) were not associated with FEV_1_. Neither personal nor central-site EC or OC was associated with FEV_1_. Personal NO_2_ exposures were significantly inversely associated with FEV_1_, at lag 0 day and almost significant at lag 1 day (*p* = 0.06). This association was stronger with a 2-day moving average of lag 0 + 1 personal NO_2_ (not shown; −1.75%; 95% CI, −2.83 to −0.673%). Central-site NO_2_ was more weakly but significantly associated with FEV_1_ deficits at lag 0, but not at lag 1 day. Although regression coefficients were negative, central-site O_3_ was not significantly associated with FEV_1_.

There was no difference in FEV_1_ associations between sexes in models including a product term of sex by air pollutant. There were also no significant interactions between use of anti-inflammatory medications and air pollutants. However, we did find significantly weaker associations among 16 children taking daily bronchodilator controller medications compared with those not taking these medications. Table 6 shows models for the relationship between percent-predicted FEV_1_ and lag 0 day air pollutants stratified by use of bronchodilator controller medications. Associations for personal NO_2_ and PM_2.5_ and ambient NO_2_ largely reflect those found among all subjects (Table 5), but are stronger in the 37 subjects not taking controller bronchodilators, including 24-hr average personal PM_2.5_. For an interquartile increase of 16.8 ppb 2-day average personal NO_2_, (not shown) percent-predicted FEV_1_ decreased by −2.45% (95% CI, −3.57 to −1.33%) in subjects not taking controller bronchodilators, but there was no association in subjects taking controller bronchodilators (*p* = 0.74).

To assess the potential importance of indoor NO_2_ sources, we retested NO_2_ models by including the presence of gas stoves as a binary variable, and a trinomial variable to account for gas stoves with or without pilot lights. Concentrations of personal NO_2_ were significantly higher for 22 subjects with gas stoves having pilot lights than for 12 subjects without gas stoves (mean = 32.4 ppb vs. 25.0 ppb, respectively), and higher than for 19 subjects with gas stoves but no pilot lights (mean = 26.4 ppb). However, gas stove covariates in the mixed models did not affect the magnitude or statistical significance of associations of FEV_1_ with personal NO_2_. In addition, stratified analyses by gas stoves did not reveal significant differences in associations between FEV_1_ with NO_2_ (*p* > 0.6). These findings held when stratified by bronchodilator group.

**Figure 2:**
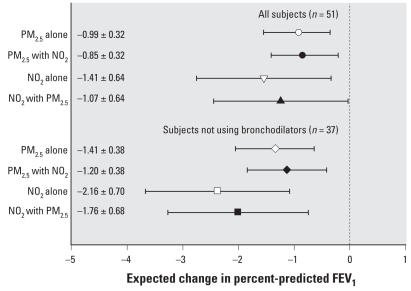
Adjusted single- and two-pollutant models (coefficient and 95% CIs) for change in FEV_1_ in relation to personal 1-hr maximum PM_2.5_ the last 24 hr, and 2-day average NO_2_ measurements. Expected change in FEV_1_ corresponds to an IQR change in the air pollutant (Table 2), and estimates are plotted by open symbols for single-pollutant models and solid symbols for models adjusting for the indicated co-pollutant. Single-pollutant models are for the subset of nonmissing observations for the other co-pollutant, and thus exclude two subjects who did not have personal PM_2.5_ data.

Figure 2 shows single-pollutant compared with two-pollutant models including subjects with both personal PM_2.5_ and NO_2_ data, and excluding the influential subject. Significant associations for 2-day average personal NO_2_ and lag 0 1-hr maximum PM_2.5_ remained when regressed together in the same model, with small decreases in estimates of association. A two-pollutant model with lag 0 24-hr averages of both personal NO_2_ and PM_2.5_ was consistent with these findings (not shown). Models testing product terms between personal NO_2_ and PM_2.5_ on FEV_1_ showed no evidence of interaction.

**Figure 3:**
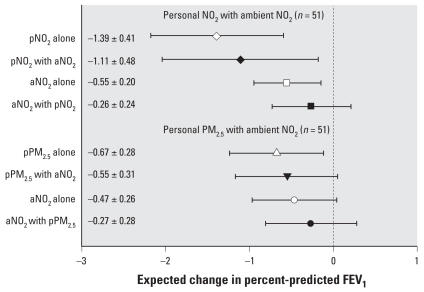
Adjusted single- and two-pollutant models (coefficient and 95% CIs) for change in FEV_1_ in relation to lag day 0 personal 24-hr average NO_2_ (pNO_2_) or PM_2.5_ (pPM_2.5_), with ambient 24-hr average NO_2_ (aNO_2_). Expected change in FEV_1_ corresponds to an IQR change in the air pollutant (Table 2), and estimates are plotted by open symbols for single-pollutant models and solid symbols for models adjusting for the indicated co-pollutant. Single-pollutant models are for the subset of nonmissing observations for the other co-pollutant in 51 subjects with pPM_2.5_ data.

We also tested two-pollutant models for 24-hr average personal NO_2_ and ambient NO_2_, and for 24-hr average personal PM_2.5_ and ambient NO_2_, excluding the influential subject. Figure 3 shows that personal NO_2_ led to a halving of the estimated FEV_1_ regression coefficient for ambient NO_2_, whereas personal NO_2_ is reduced by 20% in the two-pollutant model. Similarly, personal PM_2.5_ led to a 43% reduction in the estimated regression coefficient for ambient NO_2_ whereas the personal PM_2.5_ coefficient is reduced by 18% in the two-pollutant model. Models with maximum personal PM_2.5_ were consistent with these findings (not shown). An enhancement of FEV_1_ deficits was observed with a product term of personal NO_2_ with ambient NO_2_ (*p* < 0.06).

**Figure 4:**
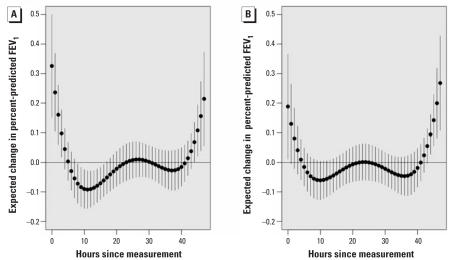
Estimated lag effect of hourly personal PM_2.5_ on FEV_1_ in the full cohort of 51 subjects. (*A*) Not adjusted for maneuver; (*B*) adjusted for maneuver. Estimates are based on a 5th-degree linear mixed-effects polynomial distributed lag model with AR(1) correlation structure. Expected change in FEV_1_ for each hour corresponds to an IQR change (21.6 μg/m^3^) in 24-hr average PM_2.5_ and estimates are plotted by solid circles. Pointwise 95% CIs are plotted by error bars. All estimates are adjusted for the previous FEV_1_ measurement, personal temperature, personal relative humidity, cumulative inhaler use on the previous day, and inhaler use during the last night, and excluding observations where there was use of inhaled as-needed bronchodilators in the preceding 4 hr.

**Figure 5:**
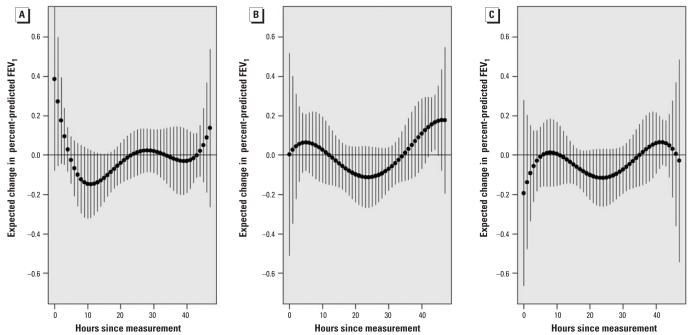
Estimated lag effect of hourly personal PM_2.5_ on FEV_1_ by session period in 37 subjects with no controller bronchodilator use. (*A*) morning; (*B*) afternoon; and (*C*) evening. Estimates are based on a 5th-degree linear mixed-effects polynomial distributed lag model with AR(1) correlation structure. Expected change in FEV_1_ for each hour corresponds to an IQR change (21.6 μg/m^3^) in 24-hr average PM_2.5_, and estimates are plotted by solid circles. Pointwise 95% CIs are plotted by error bars. All estimates are adjusted for the previous FEV_1_ measurement, personal temperature, personal relative humidity, cumulative inhaler use on the previous day, and inhaler use during the last night, and excluding observations where there was use of inhaled as-needed bronchodilators in the preceding 4 hr.

Figure 4A shows a distributed lag model across 48 hr of personal PM_2.5_ data including all 51 subjects with data. Inverse associations are shown between personal PM_2.5_ at the 9th through 18th hr preceding FEV_1_ measurements (FEV_1_ association with 9th through 18th-hr average, −0.73%; 95% CI, −1.25 to −0.22%). After 24 hr, CIs cross zero and there is evidence of a repeating 24-hr pattern across the 2 days. An unexpected positive association is shown in the 5 hr preceding FEV_1_ measurements (FEV_1_ association with 0- through 5th-hr average, 0.34%; 95% CI, −0.13 to 0.81). The 0-through 5th-hr average was confounded to −0.19% by adding an indicator for session period (morning, afternoon, or evening). This finding is attributable to morning FEV_1_ when both lung function (Table 2) and personal PM_2.5_ (Figure 1) were lowest, as expected. Thus, the positive association was temporally confounded. In contrast, the session period indicator did not confound the inverse association for the average of the 9th- through 18th-hr PM_2.5_ preceding FEV_1_. Figure 4B adjusts for session period. Figure 5A–C shows the distributed lag effects by session period in the group not taking bronchodilators (a similar but slightly less significant pattern was found using all subjects). Figure 5A shows that lags from the previous day (9th–18th hr) adversely affected morning FEV_1_ in particular. This lag effect then shifted back in time for the afternoon (Figure 5B) and evening FEV_1_ (Figure 5C) to approximately the same exposures on the previous day.

These results suggested that effects might differ by session period. Therefore, we tested product term models for session period by each pollutant. Few meaningful product terms were found at *p* < 0.1. Personal 1-hr maximum PM_2.5_ associations were stronger for the afternoon (−1.91%, *p* < 0.0001) than for the morning (−0.85%, *p* < 0.08) or evening FEV_1_ (−0.42%, *p* < 0.29). In subjects not using bronchodilators, the coefficient for personal OC was significantly more negative for afternoon FEV_1_ (−1.47%, *p* < 0.05) and morning FEV_1_ (−1.53%, *p* < 0.1) than for evening FEV_1_ (1.46%, *p* < 0.1). The coefficient for personal EC was also significantly different for morning FEV_1_ (−1.11%, *p* < 0.08). In addition, the coefficient for ambient NO_2_ lag 0 was significantly more negative for the morning FEV_1_ (−1.16%, *p* < 0.0005) than other FEV_1_ (*p* = 0.5).

## Discussion

We found that increased personal exposures to NO_2_ and PM_2.5_ were associated with lung function deficits in schoolchildren with persistent asthma. To our knowledge, this is the first report of associations between personal exposure to daily NO_2_ and FEV_1_ decrements in children with asthma. The largest magnitude of association was a 2.45% drop of percent-predicted FEV_1_ for a small interquartile increase of 16.8 ppb 2-day average NO_2_ in 37 subjects not taking controller bronchodilators. We found consistent but weaker associations for ambient NO_2_ measured at central regional sites. However, we found no associations of FEV_1_ with ambient PM, likely because of exposure error and the short sampling period of 10 days per subject.

In two-pollutant models for personal NO_2_ and PM_2.5,_ we showed considerable independence of associations with FEV_1_ suggesting that personal PM_2.5_ mass represents different causal components than personal NO_2_. This may have at least partly resulted from the different averaging times for each of the pollutants because NO_2_ was sampled over fixed 24-hr intervals, whereas PM_2.5_ was measured continuously and linked to thrice daily FEV_1_ by real time. We previously reported consistent independent associations of exhaled NO (measured once daily) with personal PM_2.5_ and NO_2_ averaged across the same 24-hr intervals in 45 of the subjects in the present analysis (Delfino et al. 2006). In addition to products of fossil fuel combustion, personal PM_2.5_ mass may also represent a variety of other exposures, including bioaerosols such as endotoxin that can exacerbate asthma. Our data also suggest that personal PM_2.5_ reflects ambient PM_2.5_, given the moderate correlation between them (*r* = 0.60).

Because of the presumed superiority of personal exposures in assessments of exposure–response relationships, we anticipated that associations for personal exposures would confound associations for ambient exposures. Furthermore, Sarnat et al. (2005) found that ambient NO_2_ concentration was a good surrogate of personal PM_2.5_ exposure. This suggests that epidemiologic findings for ambient NO_2_ may be attributable to personal PM exposures. We confirmed and expanded these expectations by finding that both personal NO_2_ and personal PM_2.5_ confounded associations of FEV_1_ with ambient NO_2_. Because personal PM_2.5_ and personal NO_2_ had largely independent effects and both confounded ambient NO_2_, they may represent both similar and different information about causal components. The interaction between personal and ambient NO_2_ further support this. In a previous panel study of children with asthma in Southern California, we also found that FEV_1_ was inversely associated with ambient NO_2_, but this was completely confounded by personal PM (Delfino et al. 2004). These findings suggest that personal PM_2.5_ and NO_2_ represent some set of causal background air pollutants also represented by ambient NO_2_. What pollutant components and sources are driving associations, though?

Outdoor NO_2_ is strongly influenced by local traffic density (Jerrett et al. 2005). Although indoor sources such as gas stoves contribute to personal exposure as well (Levy et al. 1998), we found that presence of gas stoves did not explain the association of FEV_1_ with personal NO_2_. In a large study of 482 homes in Los Angeles, outdoor home NO_2_ was well correlated with personal NO_2_ (*R*^2^ = 0.52) because of indoor infiltration (Spengler et al. 1994). Traffic-related sources of NO_2_ contribute to high spatial variability of potentially important particulate and gaseous co-pollutants (Sioutas et al. 2005). There is considerable evidence that such variability is best captured by personal exposure measurements (Jerrett et al. 2005). This is important among children who may be exposed at home, at school, and at other locations including times in vehicles. The correlation between personal and ambient NO_2_ (*r* = 0.43) as well as the confounding of the ambient NO_2_ association by personal NO_2_ are consistent with the view that in addition to local traffic sources, some part of the association we found between personal NO_2_ and FEV_1_ was attributable to ambient background sources of NO_2_. The statistical interaction between personal NO_2_ with ambient NO_2_ may reflect this source difference.

Plausible mechanisms of NO_2_ toxicity have been well described (Persinger et al. 2002) and may contribute to part of our findings. However, in experimental exposure studies of adults with mild asthma, adverse pulmonary effects of NO_2_ have generally been demonstrated at levels of exposure a magnitude higher than reported here (Kraft et al. 2005). These experimental results contrast recent epidemiologic findings showing associations of asthma outcomes in children with low levels of indoor NO_2_ (Belanger et al. 2006), of weeklong personal NO_2_ (Chauhan et al. 2003), and of ambient NO_2_ (Kim et al. 2004; Schildcrout et al. 2006). We believe the low personal NO_2_ levels we found are more likely to have served as a surrogate for traffic-related air pollutants. These pollutants may be causally related to asthmatic responses through oxidative stress responses induced by pollutants highly correlated with NO_2_ (Li et al. 2003; Seaton and Dennekamp 2003).

Given this evidence and our findings for NO_2_, it is paradoxical that we did not find FEV_1_ to be associated with particulate EC or OC in either personal or ambient samples, except, in subjects not using bronchodilators, associations of personal OC with morning and afternoon FEV_1_ and personal EC with morning FEV_1_. The carbon fraction of PM is derived primarily from products of fossil fuel combustion, so EC and OC should be reasonably good surrogates for causal pollutant components derived from those sources. In our previous report using exhaled NO, we found associations with personal and ambient NO_2_ were largely independent of associations with personal and ambient EC and OC fractions of PM_2.5_ in two-pollutant models, thus suggesting different causal pollutant components (Delfino et al. 2006). It is conceivable that volatile and semivolatile organic compounds are behind these findings given their traffic-related sources and role in particle formation (Biswas et al. 2007; Schauer and Cass 2000).

Our results for personal PM_2.5_ are consistent with recent studies showing inverse associations of personal and/or ambient PM mass with FEV_1_ among schoolchildren with asthma (Aekplakorn et al. 2003; Delfino et al. 2004; Lewis et al. 2005; Trenga et al. 2006). Magnitudes of association could not be compared, though, because of differences in both the expression of lung function effect estimates and PM measurement methods. Investigators of a recent Denver panel study failed to show associations of ambient PM_10_ with FEV_1_ in schoolchildren with persistent asthma (Rabinovitch et al. 2004), but later showed that urinary leukotriene E_4_ and rescue inhaler use during school hours were positively associated with morning average and peak PM_2.5_ (Rabinovitch et al. 2006).

Few studies of lung function in children with asthma have used personal particulate air pollution measurements, and fewer still have used real-time personal measurements that allow the assessment of effects of peak particle exposures (Delfino et al. 2004, 2006). We previously followed for 2 weeks per subject a panel of 19 children, 9–17 years of age, with persistent asthma in San Diego County (Delfino et al. 2004). We found that FEV_1_ significantly decreased similarly in relation to both 24-hr personal PM and 1-hr maximum personal PM, but FEV_1_ was not associated with outdoor ambient PM_2.5_ In the present study, we found that personal hourly peak was a stronger and more significant predictor of FEV_1_ compared with 24-hr average personal PM_2.5_.

The present associations of FEV_1_ with hourly PM_2.5_ in the distributed lag models suggest that inverse associations were primarily from exposure ≥ 8 hr before the lung function measurement. PM_2.5_ concentrations peaked in mid-morning and they were sustained for several hours into the afternoon and evening (Figure 1). Particles from morning rush hour traffic and in-vehicle exposures followed by secondary photochemical particle formation would have occurred throughout the late morning and afternoon, including time in school. Although this was possibly important in our findings, the resolution of the hourly PM_2.5_ data is limited primarily by the fact that we used fine particle mass rather than composition or other particle size fractions.

We previously conducted distributed lag analyses of hourly personal PM_2.5_ using the present panel and showed that exhaled NO (collected in the late afternoon to early evening) was positively associated with PM_2.5_ in the 5 hr before measurement (Delfino et al. 2006).

### Conclusions

The associations we found between personal NO_2_ and FEV_1_ deficits may be attributable to other more toxic pollutants from traffic-related sources. Largely independent associations between personal PM_2.5_ and FEV_1_ deficits suggest a subset of causal components different from personal NO_2_. We further conclude that associations of lung function with particulate air pollutants might be missed using ambient central-site data alone unless a large number of repeated observations per person are available. Our results may also not be generalizable to situations where central-site measurements are more representative of personal exposures in other geographic locations. Future work should focus on identifying causal pollutant components and their sources. This will require detailed assessments of exposure close to where children at risk live and attend school—a task not possible using available criteria air pollutant data.

## Figures and Tables

**Table 1:** Study group characteristics.

Characteristic	Data
Age [years, mean (range)]	13.8 (9–18)
Sex [no. (%)]	
Female	19 (35.9)
Male	34 (64.1)
Race/ethnicity no. (%)	
Hispanic^a^	26 (49.1)
White	12 (22.6)
Black	13 (24.5)
Asian	2 (3.8)
No. (%) with percent-predicted FEV_1_ < 80%^b^	18 (34.0)

a Includes 20 Hispanic subjects who gave no race and 6 who gave their race as white; two blacks and 2 Asians also gave their ethnicity as Hispanic.

b Predicted from the Third National Health and Nutrition Examination Survey (NHANES III) (Hankinson et al. 1999) from baseline spirometry.

**Table 2:** Differences in subject FEV_1_ by time of day and medication use.

Percent-predicted FEV_1_^a^	Mean ± SD	Median	Range
Overall (53 subjects)	86.8 ± 15.9	89.4	30–126
Morning	84.7 ± 17.0	88.0	33–116
Afternoon	88.6 ± 15.0	90.5	40–123
Evening	87.5 ± 15.7	89.2	30–126
Differences by medication use			
No controller medications (20 subjects)	86.3 ± 16.5	89.1	41–119
Inhaled corticosteroids (27 subjects)^b^	88.0 ± 14.4*	89.0	44–126
Antileukotrienes ± inhaled corticosteroids (13 subjects)^c^	85.2 ± 16.8*	89.2	30–126
Controller bronchodilators (16 subjects)^d^	86.1 ± 15.7	87.1	44–116

a Predicted from NHANES III (Hankinson et al. 1999) and based on data from the panel follow-up used in the present analysis.

b One subject was also using inhaled cromolyn.

c Four subjects were using antileukotrienes only, and nine were using antileukotrienes plus inhaled corticosteroids.

d Five subjects were using daily short-acting β_2_-agonist medications, two of whom were also using an anticholinergic medication (ipratropium bromide), 11 were using long-acting bronchodilator medications (sustained release theophylline and the long-acting β_2_-agonist, salmeterol xinafoate), and 14 were also using anti-inflammatory medications.

* Random-effects model *p* < 0.05 for predicted FEV1 difference from subjects not on controller medications, adjusted for study region.

**Table 3:** Descriptive statistics of daily air pollutant measurements.

Exposure	No. (missing)	Mean ± SD	Median	IQR	Min/max
Personal exposure^a^					
1-hr max PM_2.5_ (μg/m^3^)	416 (154)	90.1 ± 79.8	66.2	70.6	14.1/603.4
8-hr max PM_2.5_ (μg/m^3^)	416 (154)	46.2 ± 33.4	36.8	33.6	7.5/240.8
24-hr PM_2.5_ (μg/m^3^)	416 (154)	31.2 ± 21.8	26.0	21.6	4.3/180.0
24-hr PM_2.5_ EC (μg/m^3^)	481 (89)	0.59 ± 1.11	0.33	0.54	0/17.2
24-hr PM_2.5_ OC (μg/m^3^)	486 (84)	6.0 ± 3.4	5.2	4.3	1.0/31.5
24-hr NO_2_ (ppb)	519 (51)	28.6 ± 13.2	26.7	16.8	2.8/105.7
24-hr temperature (°C)	516 (54)	24.8 ± 3.0	25.4	4.2	17.3/32.1
Central site PM (μg/m^3^)^b^					
24-hr PM_2.5_	170 (4)	23.3 ± 17.7	17.1	15.6	2.8/87.2
24-hr PM_10_	170 (4)	45.9 ± 26.3	39.1	23.7	5.9/154.0
24-hr PM_2.5_ EC	167 (7)	1.12 ± 0.77	0.97	0.90	0.14/5.04
24-hr PM_2.5_ OC	167 (7)	5.0 ± 2.4	4.7	2.8	1.5/19.7
Central site gases (ppb)^c^					
8-hr max O_3_	174 (0)	50.7 ± 16.2	49.1	35.7	32.5/77.6
24-hr NO_2_	174 (0)	25.0 ± 3.0	25.3	6.3	19.9/29.2

Abbreviations: min, minimum; max, maximum.

a Person-days of observation, usually four personal exposure measurements per day.

b Single days of observation, which would each be linked to all four subjects followed that day.

c Around 4–5% of total hours on days with ≤ 5 contiguous hours missing were interpolated using a kernel smoother (running weighted average), including the daily calibration hour. In Riverside, 20 days with 6–24 hr of NO_2_ missing (15.3% of total days) and 1 day with 6 hr for O_3_ (0.8% of total days) were interpolated using prediction equations based on data from the nearby Rubidoux, California, station (8 km). In Whittier, 3 days with 7–24 hr of NO_2_ missing (2.4% of total days) and 1 day with 7 hr for O_3_ (0.8% of total days) were interpolated by linear regression equations based on data from the other nonmissing station data and used to estimate average regional exposure across the two stations.

**Table 4:** Exposure correlation matrix.

	Personal				Central site			
	PM_2.5_	EC	OC	NO_2_	PM_2.5_	EC	OC	NO_2_
24-hr personal PM_2.5_	1.00	0.22**	0.26**	0.38**	0.60**	0.14*	0.24**	0.32**
24-hr personal EC		1.00	0.44**	0.22**	0.02	−0.01	0.00	0.20**
24-hr personal OC			1.00	0.20**	−0.04	−0.08	0.01	0.16**
24-hr personal NO_2_				1.00	0.21**	0.20**	0.18**	0.43**
24-hr central PM_2.5_					1.00	0.51**	0.62**	0.36**
24-hr central EC						1.00	0.84**	0.61**
24-hr central OC							1.00	0.56**
24-hr central NO_2_								1.00

* *p* < 0.05, and

** *p* < 0.001, from Wald-based tests of Spearman correlation coefficients.

**Table 5:** Mixed-model estimates of the association between personal and central-site air pollutant exposures and percent-predicted FEV_1_ in 53 schoolchildren with asthma.

	Personal		Central site	
Exposure	Coefficient^a^ (95% CI)	*p*-Value	Coefficient (95% CI)	*p*-Value
PM_2.5_ 1-hr maximum				
Lag 0	−0.969 (−1.538 to −0.399)	0.001	NA	
Lag 1	0.073 (−0.595 to 0.740)	0.831	NA	
PM_2.5_ 8-hr maximum				
Lag 0	−0.801 (−1.465 to −0.137)	0.018	NA	
Lag 1	0.107 (−0.584 to 0.798)	0.761	NA	
PM_2.5_ 24-hr average				
Lag 0	−0.592 (−1.251 to 0.068)	0.079	−0.004 (−0.650 to 0.642)	0.990
Lag 1	0.049 (−0.613 to 0.711)	0.885	−0.142 (−0.775 to 0.491)	0.660
PM_2.5_ EC 24-hr average				
Lag 0	−0.080 (−0.397 to 0.238)	0.623	−0.184 (−1.038 to 0.671)	0.673
Lag 1	0.067 (−0.467 to 0.602)	0.805	−0.129 (−0.970 to 0.712)	0.763
PM_2.5_ OC 24-hr average				
Lag 0	−0.278 (−1.222 to 0.666)	0.564	−0.402 (−1.361 to 0.557)	0.411
Lag 1	−0.368 (−1.548 to 0.812)	0.540	−0.188 (−1.169 to 0.793)	0.707
NO_2_ 24-hr average				
Lag 0	−1.217 (−1.958 to −0.476)	0.001	−0.408 (−0.768 to −0.047)	0.027
Lag 1	−0.713 (−1.456 to 0.030)	0.060	−0.062 (−0.394 to 0.269)	0.712
O_3_ 8-hr maximum				
Lag 0	NA		−0.383 (−1.752 to 0.986)	0.583
Lag 1	NA		−0.904 (−2.314 to 0.506)	0.209

NA, not available. Lag 0: most recent 24-hr average measurement preceding the FEV_1_ measurement; lag 1: previous 24-hr average measurement preceding the FEV_1_ measurement.

a Coefficients represent the expected change in FEV_1_ associated with one IQR change in each air pollutant level (see Table 2), adjusted for the previous FEV_1_ measurement, personal temperature, personal relative humidity, cumulative inhaler use on the previous day, and inhaler use during the last night, and excluding observations where there was use of inhaled as-needed bronchodilators in the preceding 4 hr.

**Table 6:** Mixed-model estimates of associations between percent-predicted FEV_1_ and lag 0 air pollutant exposures stratified by preventive bronchodilator medication use.

	Not taking bronchodilator controller medications (37 subjects)		Taking bronchodilator controller medications (16 subjects)	
Exposure	Coefficient^a^ (95% CI)	*p*-Value	Coefficient (95% CI)	*p*-Value
Personal				
PM_2.5_ 1-hr maximum	−1.324 (−2.001 to −0.648)	0.0001	−0.145 (−1.230 to 0.940)	0.792
PM_2.5_ 24-hr average	−0.785 (−1.526 to −0.043)	0.038	0.004 (−1.478 to 1.486)	0.996
PM_2.5_ EC	−0.249 (−1.022 to 0.524)	0.527	−0.075 (−0.442 to 0.293)	0.689
PM_2.5_ OC	−0.577 (−1.636 to 0.482)	0.285	0.441 (−1.678 to 2.561)	0.682
NO_2_	−1.443 (−2.257 to −0.629)	0.001	−0.587 (−2.432 to 1.257)	0.531
Central site				
PM_2.5_	−0.003 (−0.719 to 0.712)	0.992	−0.101 (−1.745 to 1.544)	0.904
PM_2.5_ EC	−0.616 (−1.659 to 0.428)	0.247	0.733 (−0.921 to 2.387)	0.383
PM_2.5_ OC	−0.503 (−1.666 to 0.660)	0.396	−0.329 (−2.198 to 1.540)	0.729
NO_2_	−0.555 (−0.966 to −0.143)	0.008	−0.048 (−0.859 to 0.764)	0.908

Lag 0: most recent 24-hr average measurement preceding the FEV_1_ measurement.

a Coefficients represent the expected change in FEV_1_ associated with one IQR change in each air pollutant level (see Table 2), adjusted for the previous FEV_1_ measurement, personal temperature, personal relative humidity, cumulative inhaler use on the previous day, and inhaler use during the last night, and excluding observations where there was use of inhaled as-needed bronchodilators in the preceding 4 hr.
